# Power law of path multiplicity in complex networks

**DOI:** 10.1093/pnasnexus/pgae228

**Published:** 2024-06-05

**Authors:** Ye Deng, Jun Wu

**Affiliations:** Department of Systems Science, Faculty of Arts and Sciences, Beijing Normal University, Zhuhai 519087, China; International Academic Center of Complex Systems, Beijing Normal University, Zhuhai 519087, China; Department of Systems Science, Faculty of Arts and Sciences, Beijing Normal University, Zhuhai 519087, China; International Academic Center of Complex Systems, Beijing Normal University, Zhuhai 519087, China

**Keywords:** complex networks, path multiplicity, power law, hesitant world, Path Hesitation Index

## Abstract

Complex networks describe a wide range of systems in nature and society. As a fundamental concept of graph theory, the path connecting nodes and edges plays a vital role in network science. Rather than focusing on the path length or path centrality, here we draw attention to the path multiplicity related to decision-making efficiency, which is defined as the number of shortest paths between node pairs and thus characterizes the routing choice diversity. Notably, through extensive empirical investigations from this new perspective, we surprisingly observe a “hesitant-world” feature along with the “small-world” feature and find a universal power-law of the path multiplicity, meaning that a small number of node pairs possess high path multiplicity. We demonstrate that the power-law of path multiplicity is much stronger than the power-law of node degree, which is known as the scale-free property. Then, we show that these phenomena cannot be captured by existing classical network models. Furthermore, we explore the relationship between the path multiplicity and existing typical network metrics, such as average shortest path length, clustering coefficient, assortativity coefficient, and node centralities. We demonstrate that the path multiplicity is a distinctive network metric. These results expand our knowledge of network structure and provide a novel viewpoint for network design and optimization with significant potential applications in biological, social, and man-made networks.

**Significance Statement**

This study offers a new perspective in network science by exploring path multiplicity, a metric that measures the diversity of routing choices in complex networks. Our research uncovers a “hesitant-world” feature alongside the well-known “small-world” effect, revealing a universal power law in path multiplicity. This finding suggests that a few node pairs have a high number of shortest paths, a pattern more pronounced than the scale-free property of node degree. These insights challenge traditional network models and highlight the unique role of path multiplicity as a network metric. Our work provides fresh viewpoints for network design and optimization, with broad implications for biological, social, and engineered systems, enhancing our understanding of network structures.

## Introduction

Complex networks, ubiquitous in both natural and engineered systems, play a vital role in understanding the underlying structural and functional characteristics of various phenomena (1, 2). These networks, spanning diverse realms including social networks (3, 4), biological networks (5, 6), transportation networks (7, 8), power grids (9, 10), are intrinsically sophisticated, involving myriads of interconnections and interactions among entities. Despite their widespread and distinct manifestations, complex networks share certain universal topological features, such as the small-world effect (11, 12) and the scale-free property (13, 14), which have been well-documented in the literature. The effort to develop a universal view of complex networks and to devise ways of using knowledge of network structure to understand, control, or design system behavior has generated both excitement and confusion (15), and over the past decades there have been numerous advances in the field of network science (16–29).

As we know, a network is composed of nodes and edges. In graph theory, a path is defined as a unique, ordered sequence of nodes and edges wherein each node is adjacent to its successive node, connected through edges without revisiting any node (30, 31). As a basic concept of network science, the study of path has gained much attention for a long time (32–38). Among these studies, the most remarkable and widely discussed topic is the small-world effect (39, 40). It’s shown that in many—perhaps most—networks the average shortest path lengths between nodes are surprisingly small and typically scale as a logarithm function with the network size. Moreover, much attention has been paid to the path centrality (41–51), known as the betweenness that measures the extent to which a node (edge) lies on paths between other node pairs (52). However, few studies have explored the problem of path multiplicity.

Suppose that we need to find a path between a node pair, in most situations, there are several alternative paths of different lengths and one would choose the shortest one. If there is only one shortest path, things become simple. But when multiple shortest paths are available, one would be hesitant. It has been shown that excessive choices may cause “choice overload” or “decision paralysis” (53, 54) and then affect the efficiency of decision-making. Despite the importance of the path multiplicity, actual situations in the real world remain undiscovered: it is a “hesitant-world” or a “decisive-world”? In this study, we define the path multiplicity as the number of shortest paths between node pairs. We aim to investigate the probability distributions of path multiplicity in real-world networks and then explore the relationship between the path multiplicity and existing typical network metrics.

## Results

### Definition of path multiplicity

Complex networks can be formed as a simple undirected graph G(V,E), in which *V* is the node set, E⊆V×V is the edge set. Let N=|V| be the number of nodes and W=|E| be the number of edges, respectively. Denote by $A(G)=(a_{ij}{)}_{N\times N}$ the adjacency matrix of *G*, where $a_{ij}=a_{\;ji}=1$ if $v_{i}$ and $v_{j}$ are connected, and $a_{ij}=a_{\;ji}=0$ otherwise. Let $d_{i}$ be the degree of node $v_{i}$, i.e. $d_{i}=\sum_{\;j=1}^{N}a_{ij}$. The edge density of a network is given by $p_{\mathrm{edge}}=\langle k\rangle /(N-1)$, where ⟨k⟩ is the average degree of the network. A path $P=v_{i}e_{1}v_{1}e_{2}\cdots e_{k}v_{j}$ is an alternating sequence of nodes and edges without any repetition of nodes, and then the length of *P* is defined as the number of edges in the path. Denote by $l_{ij}$ the length of the shortest path from node $v_{i}$ to $v_{j}$. Denote by $h_{ij}$ the number of shortest paths from node $v_{i}$ to $v_{j}$ (see Methods for a fast algorithm). In this article, we only focus on the simple connected graph and thus $h_{ij}=h_{\;ji}\geq 1$ if i≠j and $h_{ii}=0$.

Consider that a larger value of $h_{ij}$ would make the routing choice more hesitant, here we call $h_{ij}$ the Path Hesitation Amount (PHA) of a node pair $\left(v_{i},v_{j}\right)$ and then denote by $H(G)=(h_{ij}{)}_{N\times N}$ the Path Hesitation Matrix (PHM). To quantify the path multiplicity of a node $v_{i}$, we define the average PHA of all node pairs associated with $v_{i}$ as the Path Hesitation Degree (PHD) of $v_{i}$, which can be written as


(1) $$\tilde{d_{i}}=\frac{1}{N-1}\sum_{\;j\neq i}h_{ij}.$$

To characterize the path multiplicity of the entire network, we take the average of PHD values of all nodes (or the average of PHA values of all node pairs) and call it the Path Hesitation Index (PHI), which can be shown as


(2) $$\Phi (G)=\frac{1}{N}\sum_{i=1}^{N}\tilde{d_{i}}=\frac{\sum_{i=1}^{N}\sum_{\;j\neq i}h_{ij}}{N(N-1)}.$$

**Fig. 1.**
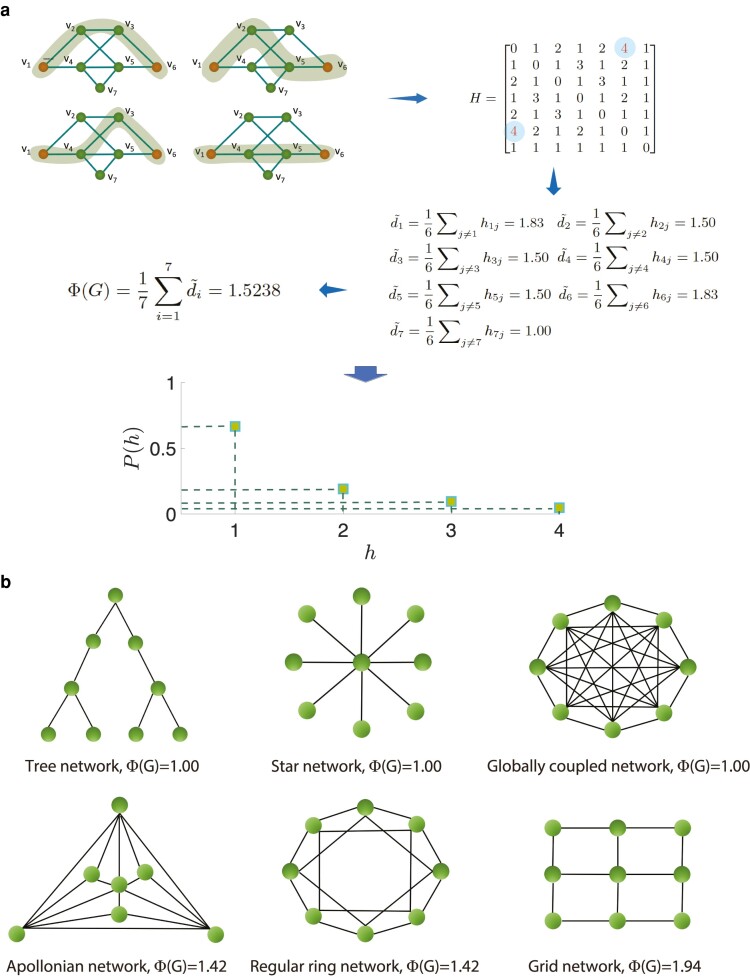
Illustration of the PHA, PHD, and PHI. a) In an example network with 7 nodes and 10 edges, darker shaded areas denote 4 shortest paths of length 3 between the node pair ($v_{1}$, $v_{6}$), namely $v_{1}$–$v_{2}$–$v_{3}$–$v_{6}$, $v_{1}$–$v_{2}$–$v_{5}$–$v_{6}$, $v_{1}$–$v_{4}$–$v_{3}$–$v_{6}$, and $v_{1}$–$v_{4}$–$v_{5}$–$v_{6}$. It leads to that the PHA of the node pair ($v_{1}$, $v_{6}$) is 4. Based on the PHM *H*, we can calculate the PHD $\tilde{d_{i}}$ of each node, respectively. Consequently, the PHI for the example network can be obtained as Φ(G)=1.5238. The probability distributions of PHA is also shown. There are 14 node pairs (66.67%) with the PHA value h=1, four node pairs (19.04%) with the PHA value h=2, two node pairs (9.52%) with the PHA value h=3, and one node pair (4.77%) with the PHA value h=4. b) The PHI values of classical network topologies.

It’s easy to know that Φ(G)≥1. A large value of this global network metric *Φ* implies that it is a “hesitant-world.” Conversely, it is a “decisive-world.” Figure 1a illustrates an example of the calculation of PHA, PHD, and PHI proposed in this article. Furthermore, Fig. 1b shows the PHI values of classical network topologies. We see that, for tree network, star network, and globally coupled network, there is only one shortest path between all node pairs. Thus, the PHI of these networks are equal to 1, meaning that they are “decisive-world.” For apollonian network, regular ring network, and grid network, there may be several shortest paths between node pairs, which implies a “hesitant-world.”

### Empirical results

**Fig. 2.**
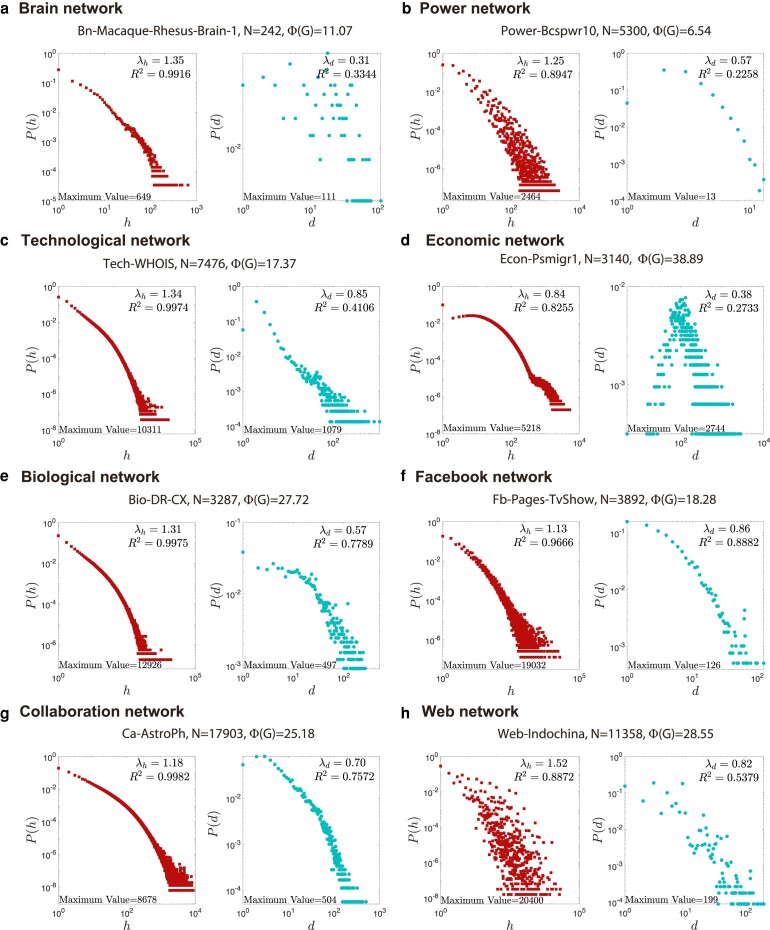
PHA distributions P(h) and degree distributions P(d) on log–log scales of eight real-world networks. The goodness of fit $R^{2}$ and the maximum value of each distribution are also presented.

To investigate the path multiplicity in the real world, we collected 32 typical real-world networks of 8 categories that have a wide coverage of technological, social, biological, and economic domains, with their sizes ranging from hundreds to tens of thousands of nodes. Considering that real-world networks may be disconnected, we implement experiments on the giant connected component of each real-world network. In Fig. 2, we show the PHA distribution P(h) along with the degree distribution P(d) for 8 networks out of these 32 networks. The metadata and results for all 32 networks are in Supplementary Materials.

From the empirical results shown in Fig. 2, we observe that most degree distributions follow a power-law distribution, i.e. $P(d)∼d^{-\lambda_{d}}$, which is a well-known fact and suggests that there are a small number of nodes with high degree. Moreover, we surprisingly discover that the PHA distribution also follows a stronger power-law distribution in almost all networks, i.e. $P(h)∼h^{-\lambda_{h}}$. It means that, distinct from the bell-shaped distribution, PHA exhibits a long-tail in which a small proportion of node pairs have an extraordinarily large number of shortest paths. For instance, in the Web-Indochina network with 11,358 nodes shown in Fig. 2h with $\lambda_{h}=1.52$ and $\lambda_{d}=0.82$, the maximum value of PHA is 20,400. Even in the Bn-Macaque-Rhesus-Brain-1 brain network with only 242 nodes shown in Fig. 2a, the maximum PHA value also reaches to 649. We further conduct statistical analysis of full range fitting (Supplementary Materials have details). The results show that the goodness of fit $R^{2}$ for the power law of P(h) is much better than the goodness of fit for the power law of P(d). The goodness of fit for the power law of P(h) is almost all exceed 0.9 in 32 real-world networks; however, the goodness of fit for the power law of P(d) is mostly much lower than 0.9. It is noteworthy that, despite in cases where the degree distribution significantly deviates from a power law, as exemplified by the Bn-Macaque-Rhesus-Brain-1 network in Fig. 2a ($R^{2}=0.3344$) and the Econ-Psmigr1 network in Fig. 2d ($R^{2}=0.2733$), the PHA still adheres to a power law distribution, with corresponding $R^{2}$ values of 0.9916 and 0.8255, respectively.

Moreover, along with the power-law distribution for PHA, we observe that the global network metric PHI is also generally greater than expected. As shown in Fig. 2d, the PHI of the Econ-Psmigr1 network with 3,140 nodes reaches to a staggering value of 38.89, which means that on average each node pair has 38.89 shortest paths. Even in small size networks, such as the Bn-Macaque-Rhesus-Brain-1 brain network with only 242 nodes shown in Fig. 2a, the PHI also reaches to 11.07. These findings reveal that the real world emerges a “hesitant-world” feature. In the real world, the well-known “small-world” effect indicates that the length of shortest path between node pair is relatively short, which is linked to the connection efficiency. Here, the “hesitant-world” feature implies that the number of shortest path between node pair is relatively large, which is linked to the decision-making efficiency. As far as we know, this is an undiscovered and neglected phenomenon in the field of network science.

### Model simulation

**Fig. 3.**
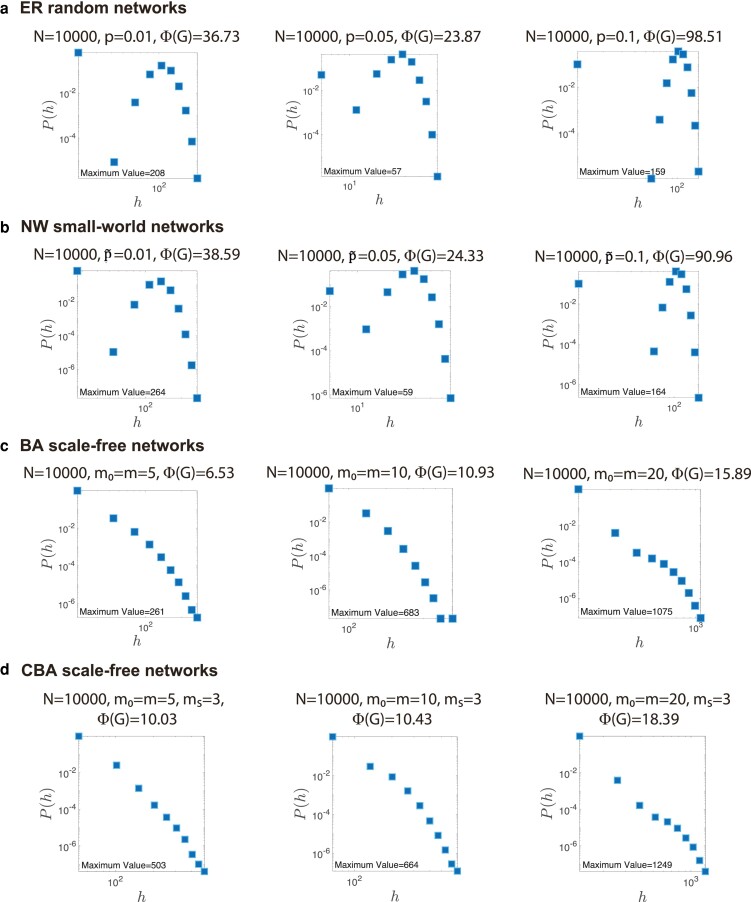
Path multiplicity in typical model networks. a) ER random networks, where *p* denotes the probability of edges between nodes. b) NW small-world networks, where $\tilde{\;p}$ denotes the rewiring probability. c) BA scale-free networks, where $m_{0}$ denotes the initial number of nodes and *m* denotes the number of edges added in each step. d) Clustered BA scale-free networks, which extend the standard BA scale-free network model to include a “triad formation step,” where $m_{s}$ denotes the number of edges added to neighbors of each preferentially attached node.

In the past decades, many classic network models have been proposed to characterize network structures in the real world. To validate whether these typical network models can also capture the “hesitant-world” feature and the power-law characteristic of path multiplicity, we investigate the path multiplicity of three classical model networks: Erdős–Rényi (ER) random networks (55), Newman–Watts (NW) small-world networks (12), Barabási–Albert (BA) scale-free networks (14), and Cluster Barabási–Albert (CBA) scale-free networks (56). The experimental results are shown in Fig. 3.

**Fig. 4.**
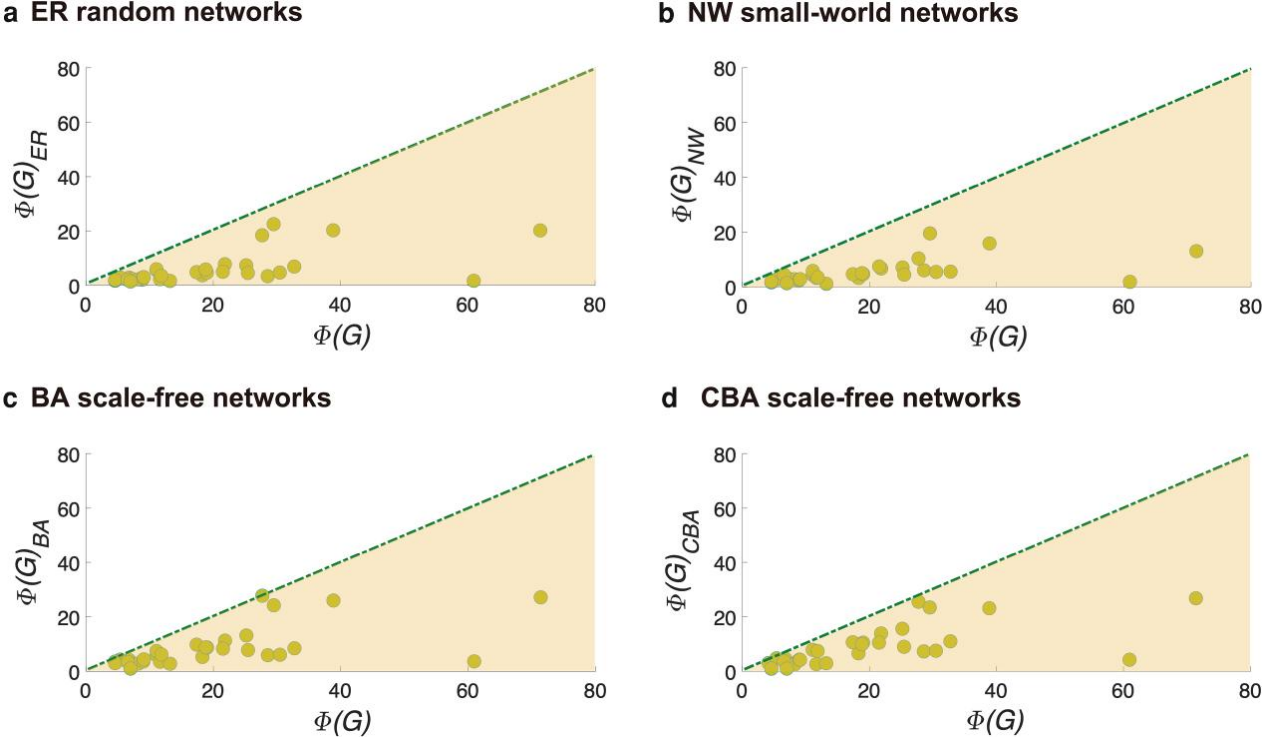
Scatter plots for Φ(G) of 32 real networks and corresponding model networks $G_{\mathrm{ER}}$, $G_{\mathrm{NW}}$, $G_{\mathrm{BA}}$ and $G_{\mathrm{CBA}}$ with the same network size and similar edge density. The dashed line and the darker shaded area denote a reference slope of 1. a) ER random networks, b) NW small-world networks, c) BA scale-free networks, and d) CB scale-free newworks.

From Fig. 3a–d, we find that, ER random networks and NW small-world networks obviously deviate from power-law distributions. Even though BA networks and CBA networks exhibit a power-law-like PHA distributions, the maximum value of PHA is much smaller than the maximum values of PHA in real networks with similar network size. It suggests that these typical model networks could not recall the path multiplicity observed in real-world. In order to further explore the differences between the model networks and the real-world networks, we show in Fig. 4 the PHI values of 32 real-world networks along with the PHI values of reference model networks with the same network size and similar edge density. We find that PHI values of model networks are significantly lower than PHI values of real-world networks. It emphasizes that the “hesitant-world” feature and the power law of the path multiplicity in complex networks are new findings that cannot be explained by existing network theories. It also suggests that there are neglected underlying mechanisms of the formation of real-world networks.

### Relationship between path multiplicity and other network metrics

**Fig. 5.**
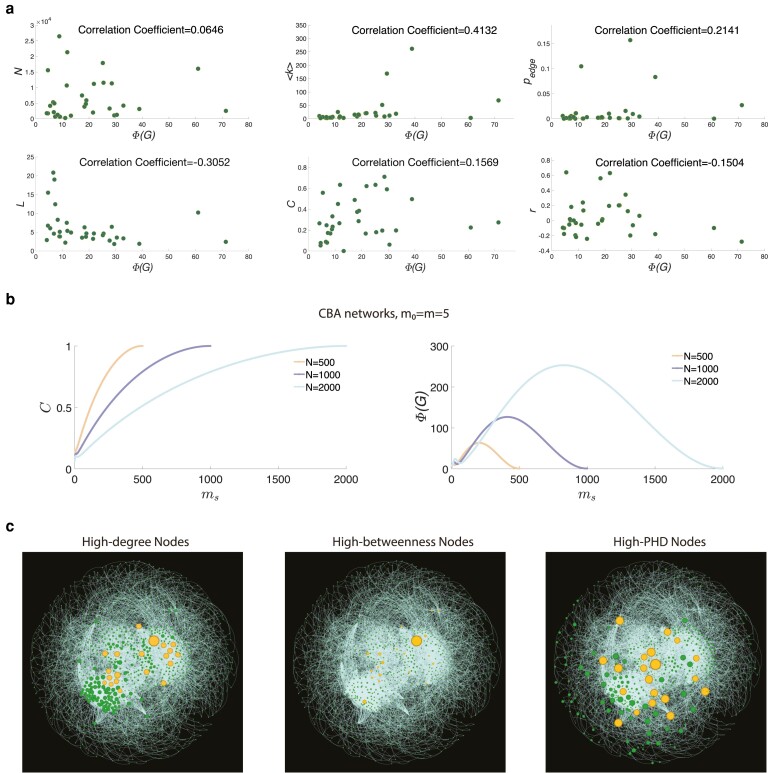
The relationship between the path multiplicity and typical network metrics. a) Scatter plots along with the correlation coefficients for Φ(G) and the number of nodes *N*, the average degree ⟨k⟩, the edge density $p_{\mathrm{edge}}$, the assortativity coefficient *r*, the clustering coefficient *C*, and the average shortest path length *L* in 32 real networks. b) The impact of clustering coefficient on the path multiplicity in the CBA network model, where $m_{s}$ denotes the number of edges added to neighbors of each preferentially attached node. Specifically, the newly added node would connect to all possible neighbors if there remains not enough neighbors in the “triad formation step.” On the left part, we show the clustering coefficient *C* as a function of $m_{s}$ with different network sizes. On the right part, we show the PHI Φ(G) as a function of $m_{s}$ with different network sizes. c) Critical nodes based on degree centrality, betweenness centrality and PHD in Bn-macaque-rhesus-brain network. The size of each node is proportional to metric values. Top 20 critical nodes based on each metric are emphasized in figures.

With the development of network science, a variety of useful quantities or measures that capture particular features of the network topology have been proposed. We present in Fig. 5 the relationship between the path multiplicity and existing network metrics. First, the PHI Φ(G) compared to 6 typical global network metrics in 32 real-world networks are shown in Fig. 5a using scatter plots. We observe that the correlation coefficients are generally lower than 0.5. It means that there does not exist a significant correlation between the path multiplicity and other network metrics.

To explore the impact of network properties on the path multiplicity in depth, we change the classical clustering coefficient by using the CBA network model, and observe how the path multiplicity changes. In Fig. 5b, we find that along with increasing values of clustering coefficient, the PHI exhibit a complex bimodal tendency. Moreover, from a microscopic perspective, we display in Fig. 5c the critical nodes based on the degree centrality, the betweenness centrality and the PHD in the Bn-macaque-rhesus-brain network, respectively. It is easy to see that the overlap between the high PHD nodes and the high degree (or betweenness) nodes is quite low. A large number of nodes with high PHD values do not necessarily have large degree (or betweenness). It implies that the path multiplicity is a distinctive network metric.

## Conclusion

In this article, we have investigated the problem of path multiplicity, which is fundamental and neglected in the study of network science. We first introduced the concept of PHA $h_{ij}$ based on the number of shortest paths between a node pair, which relates to the routing choice diversity and decision-making efficiency. Based on PHA, we defined the PHD $\tilde{d}$ to describe the path multiplicity of each node. Furthermore, we defined the PHI Φ(G) to characterize the path multiplicity of the entire network. Utilizing these metrics, we have studied the path multiplicity in real-world networks. Surprisingly, we discovered a universal power-law distribution for PHA, i.e. $P(h)∼h^{-\lambda_{h}}$. Unlike the expected bell-shaped distribution, we found a long-tail distribution which suggests that a small proportion of node pairs have an extraordinarily large number of shortest paths. As we know, the degree distribution exhibits power-law behavior in the real-world networks, which is known as the scale-free property. We demonstrated that the power law of P(h) is much stronger than the power law of node degree. The goodness of fit for the power law of P(h) is almost all exceed 0.9. Then we observed that the global network metric PHI is also generally greater than expected. These findings reveal that the world we live in is not only a “small-world” but also a “hesitant-world.” Rather than the “small-world” effect related to the connection efficiency, here the “hesitant-world” feature is linked to the decision-making efficiency. The “small-world” effect indicates that most nodes can be reached from any other by a small number of steps, despite the large size of the network, however, the “hesitant-world” effect implies that we may be hesitant among a large number of choices, despite the small size of the network. Moreover, we have investigated in depth the difference between empirical results and simulation results, and the difference between the path multiplicity and other network metrics. It was demonstrated that typical model networks could not reproduce the “hesitant-world” feature, and the path multiplicity is a brand-new network metric.

Human nature is to seek optimal solutions. When we navigate from one node to another, the preference is to identify the shortest path. However, the presence of path multiplicity, while broadening choices, might lead to hesitancy in decision-making. The research of path multiplicity in complex networks is a fundamental issue with significant potential applications in numerous domains. We hope that our work will stimulate further studies. For instance, what is the underlying mechanism of the “hesitant-world” feature? How does the “hesitant-world” feature influence the functionality and dynamic behavior of networks? What is the optimal network topology based on the path multiplicity? Although the problem of path multiplicity has not received much attention, we believe that it will turn out to be widespread in biological, social and man-made systems, such as (i) planning city critical infrastructures; (ii) understanding structure and function of brain networks; (iii) building efficient global supply chain networks; (iv) optimizing deep neural networks; (v) analyzing social structure and organizations.

The “hesitant-world” feature provides a novel concept through which to view and analyze the structure of complex networks. This concept emphasizes the psychological and practical challenges posed by multiple optimal paths. By introducing the idea of hesitation in decision-making within networks, it highlights how individuals or entities may struggle with choosing between equally beneficial paths. It enriches the dialog between network science and decision theory, offering a multidisciplinary approach to understanding and designing better networked systems.

## Methods

### Fast algorithm for the number of shortest paths

Generally, we can calculate shortest path lengths between all node pairs using the well-known Floyd algorithm with a time complexity of $O(N^{3})$. However, the Floyd algorithm cannot provide numbers of shortest paths between node pairs. Moreover, although the Breadth-First Search or Depth-First Search can present all paths between a node pair with a time complexity of $O(N^{2})$, the time complexity will be $O(N^{4})$ if we need to calculate numbers of shortest paths between all node pairs.

Path multiplicity mainly concerned about the number of shortest paths between a node pair, rather than specific nodes traversed by a shortest path. In this article, we adopt a fast algorithm for numbers of shortest paths to simultaneously compute numbers and lengths of shortest paths between all node pairs in a network. The algorithm is as follows.

**Step 0**. Initialize the PHM $H=(h_{ij}{)}_{N\times N}$ and the Path Length Matrix $L=(l_{ij}{)}_{N\times N}$, i.e. $h_{ij}=h_{\;ji}=0$ and $l_{ij}=l_{\;ji}=0$. Let k=1.

**Step 1**. If $h_{ij}=h_{\;ji}\neq 0,\;\forall \;i\neq j$, the algorithm terminates. Otherwise, (i) Calculate the transition matrix $T=(t_{ij}{)}_{N\times N}$, where $t_{ij}=0$ if $h_{ij}\neq 0$, $t_{ij}=1$ if $h_{ij}=0$; (ii) Update *H* by $H=H+T⊙A^{k}$, where ⊙ represents the Hadamard product; (iii) Update *L* by L=L+k⋅J, where the matrix $J=I(T⊙A^{k}\neq 0)$.

**Step 2**. Let k=k+1. Go to Step 1.

### Parameter estimation of power-law distribution

Mathematically, a quantity *x* obeys a power law if it is drawn from a probability distribution with a density of the form


$$p(x)\propto x^{-\alpha },$$

where *α* is a constant parameter of the distribution known as the exponent or scaling parameter.

To obtain the parameter estimation for power-law distribution of empirical data, we adopt the Power Fitting toolbox proposed by Matlab R2022a. The detailed information about the toolbox can be found through https://www.mathworks.com/help.

### The goodness of fit $R^{2}$

Let $y=[y_{1},y_{2},\ldots ,y_{n}{]}^{T}$ be the vector of observed data values, and let $f=[f_{1},f_{2},\ldots ,f_{n}{]}^{T}$ be the vector of predicted values from a model. Define the residuals as e=y−f, with elements $e_{i}=y_{i}-f_{i}$ for i=1,2,…,n.

The mean of the observed data is given by $\bar{y}=\frac{1}{n}\sum_{i=1}^{n}y_{i}.$ The total sum of squares, which measures the total variance in the observed data, is defined as


(3) $${\mathrm{SS}}_{\mathrm{tot}}=\sum_{i=1}^{n}(y_{i}-\bar{y}{)}^{2}.$$

The residual sum of squares, which measures the variance in the residuals, is defined as


(4) $${\mathrm{SS}}_{\mathrm{res}}=\sum_{i=1}^{n}e_{i}^{2}=\sum_{i=1}^{n}(y_{i}-f_{i}{)}^{2}.$$

The coefficient of determination, $R^{2}$, is then defined as:


(5) $$R^{2}=1-\frac{{\mathrm{SS}}_{\mathrm{res}}}{{\mathrm{SS}}_{\mathrm{tot}}}.$$

In the best case, the modeled values exactly match the observed values, which results in $SS_{\mathrm{res}}=0$ and $R^{2}=1$.

## Supplementary Material

pgae228_Supplementary_Data

## Data Availability

The network datasets used are available via https://www.networkrepository.com/index.php. The algorithm codes can be found through https://github.com/gituserbnu/shortestpath.git.
