# Supplementary material for: Power law of path multiplicity in complex networks
Source: PNAS Nexus. 2024 Jun 5;3(6):pgae228. doi: 10.1093/pnasnexus/pgae228 (PMC11184978; doi:10.1093/pnasnexus/pgae228)
Supplement: pgae228_Supplementary_Data [file pgae228_supplementary_data.pdf]

PNAS nexus

Supporting Information for

Power-law of path multiplicity in complex networks

Ye Deng, Jun Wu

Jun Wu.
E-mail: junwu@bnu.edu.cn

This PDF file includes:

Figs. S1 to S8
Table S1

Brain networks

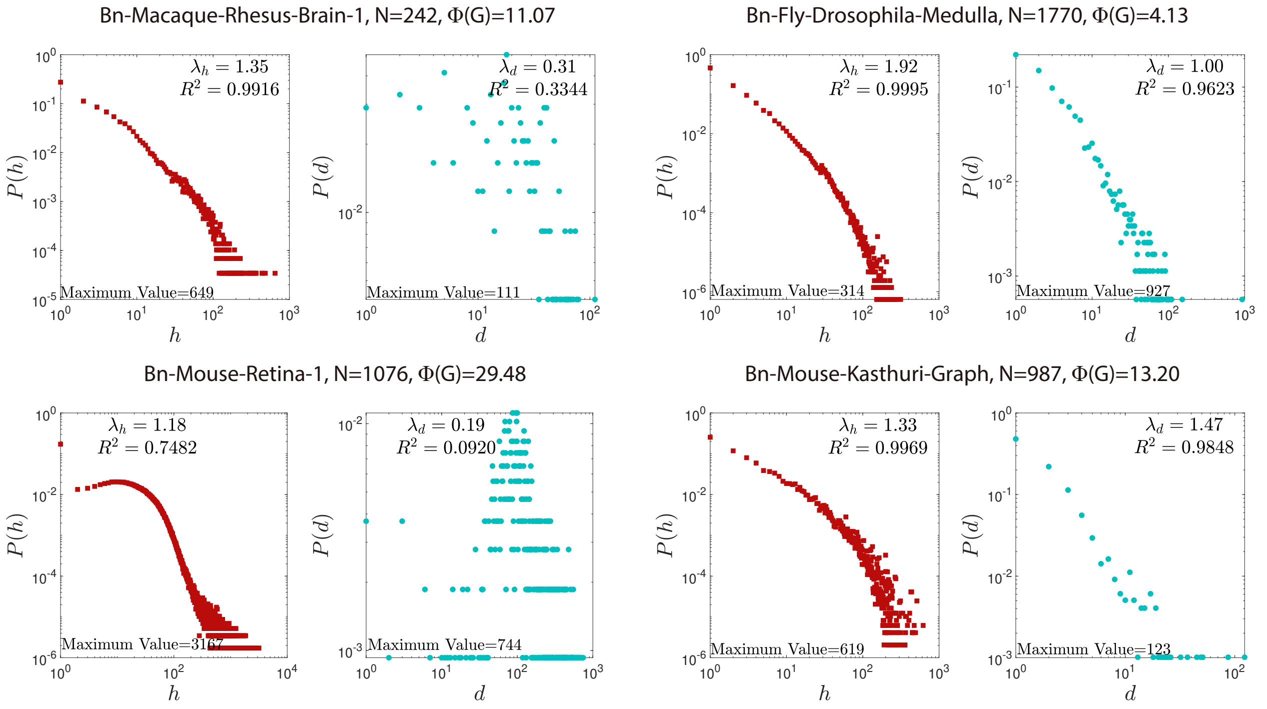

Fig. S1. PHA distributions $P(h)$ (red square) and degree distributions $P(d)$ (green circle) on log-log scales of brain networks. The goodness of fit R^2 and the maximum value of each distribution are also presented.

Power networks

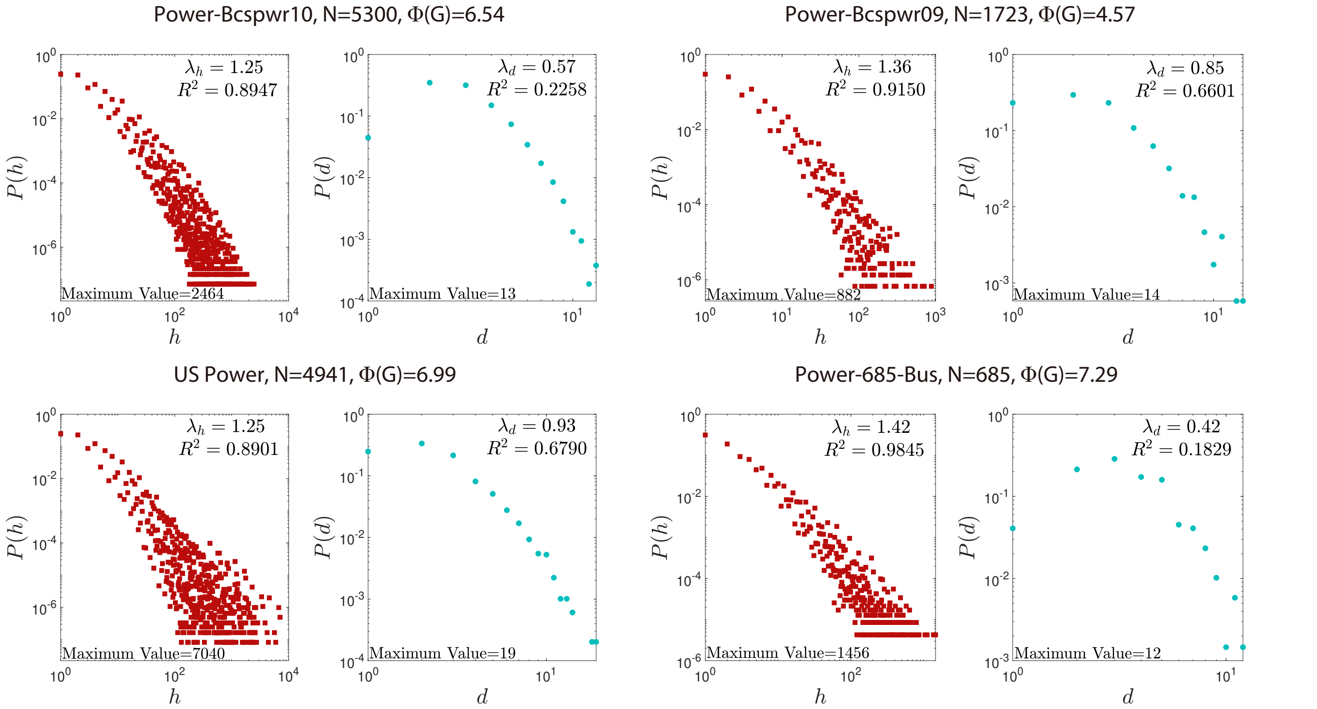

Fig. S2. PHA distributions $P(h)$ (red square) and degree distributions $P(d)$ (green circle) on log-log scales of power networks. The goodness of fit R^2 and the maximum value of each distribution are also presented.

Technological networks

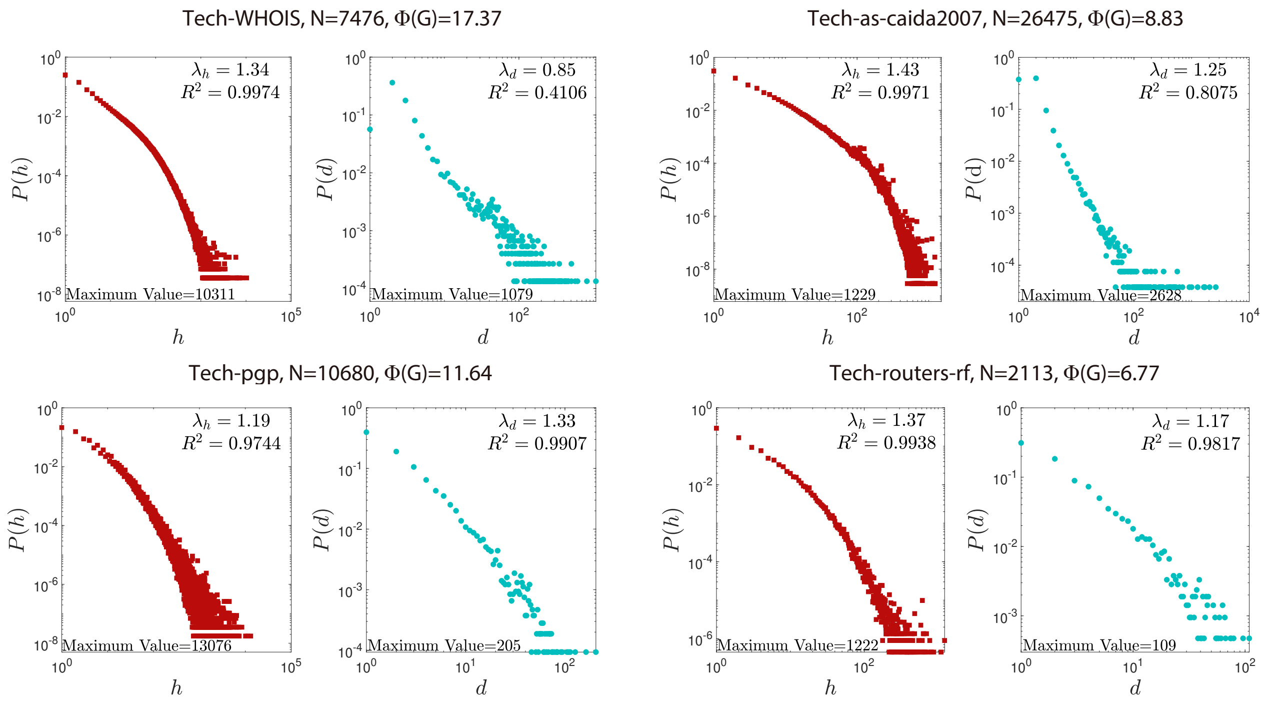

Fig. S3. PHA distributions $P(h)$ (red square) and degree distributions $P(d)$ (green circle) on log-log scales of technological networks. The goodness of fit R^2 and the maximum value of each distribution are also presented.

Economic networks

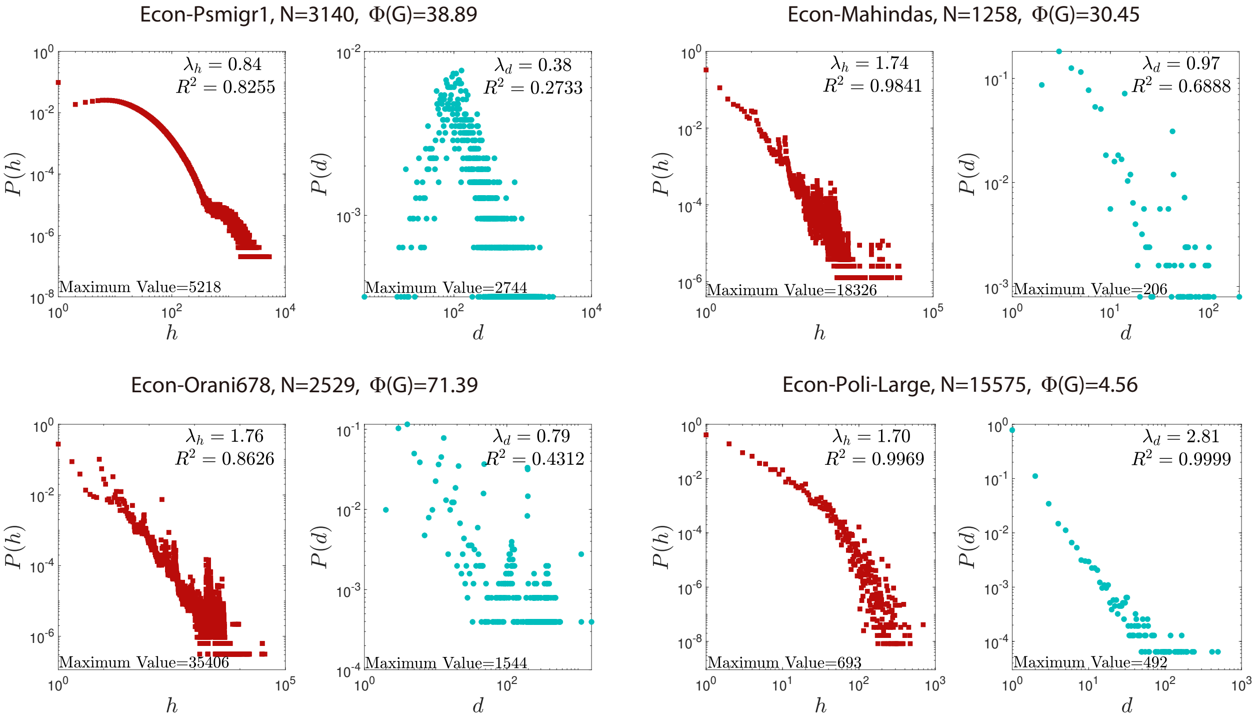

Fig. S4. PHA distributions $P(h)$ (red square) and degree distributions $P(d)$ (green circle) on log-log scales of economic networks. The goodness of fit R^2 and the maximum value of each distribution are also presented.

Biological networks

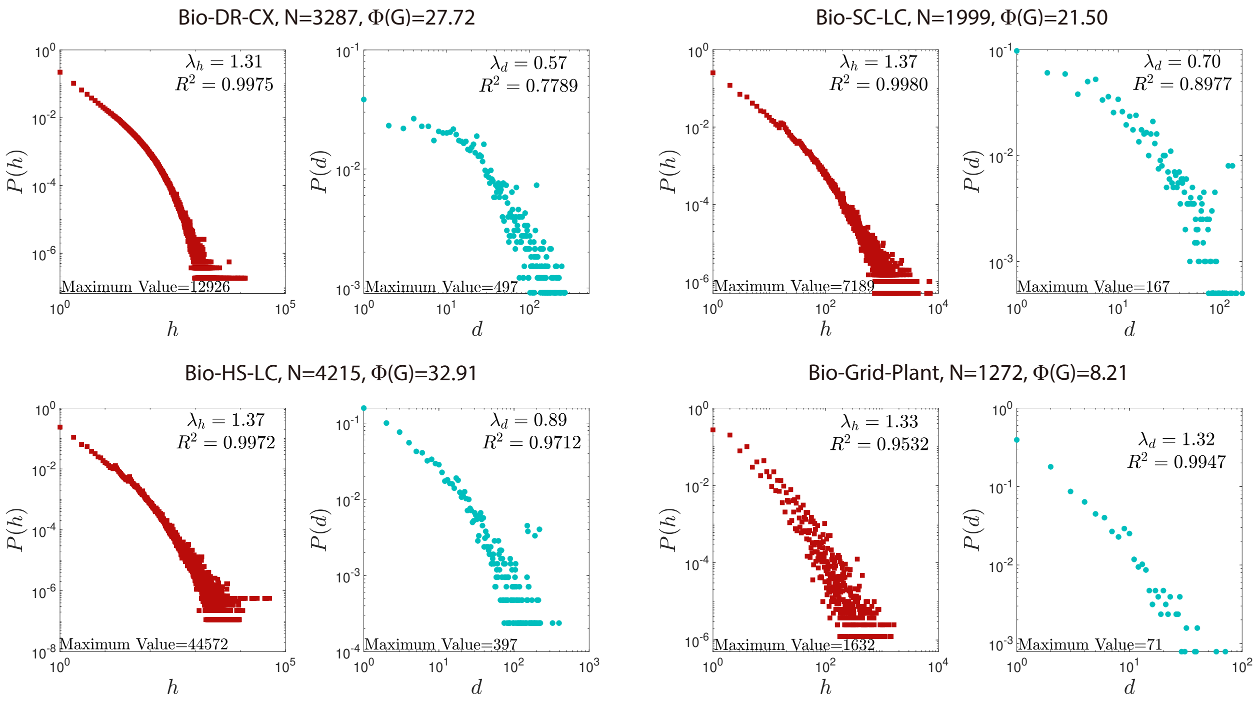

Fig. S5. PHA distributions $P(h)$ (red square) and degree distributions $P(d)$ (green circle) on log-log scales of biological networks. The goodness of fit R^2 and the maximum value of each distribution are also presented.

Facebook networks

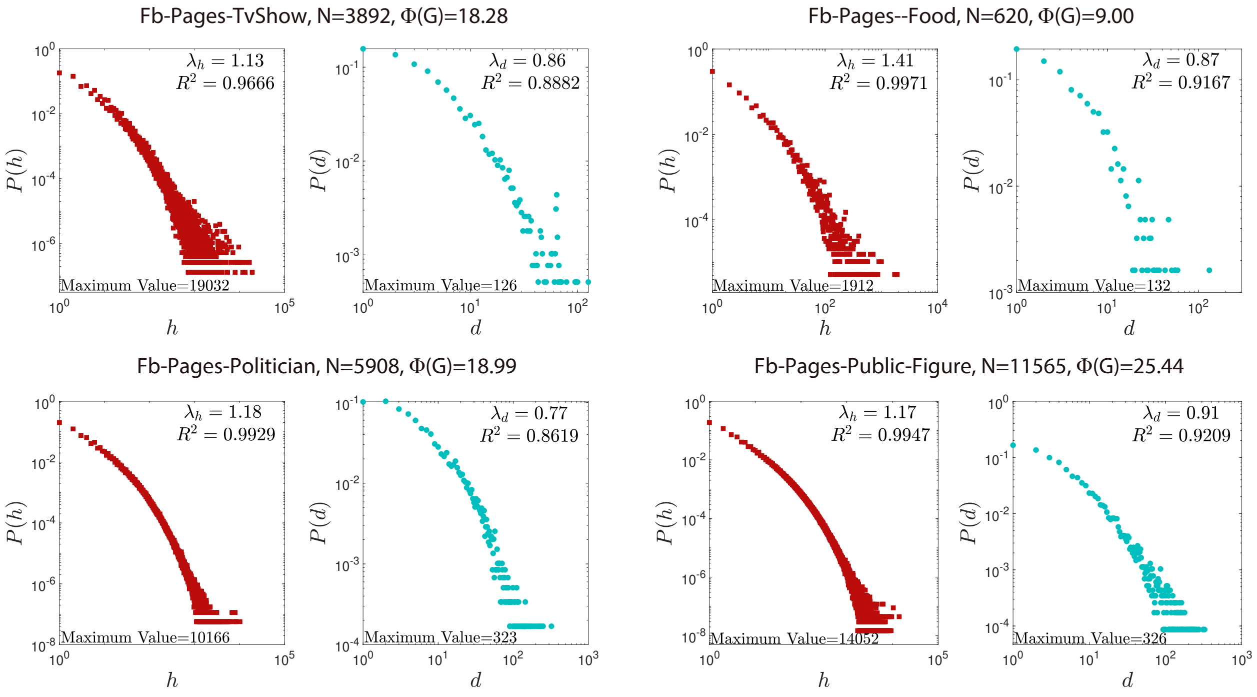

Fig. S6. PHA distributions $P(h)$ (red square) and degree distributions $P(d)$ (green circle) on log-log scales of facebook networks. The goodness of fit R^2 and the maximum value of each distribution are also presented.

Collaboration networks

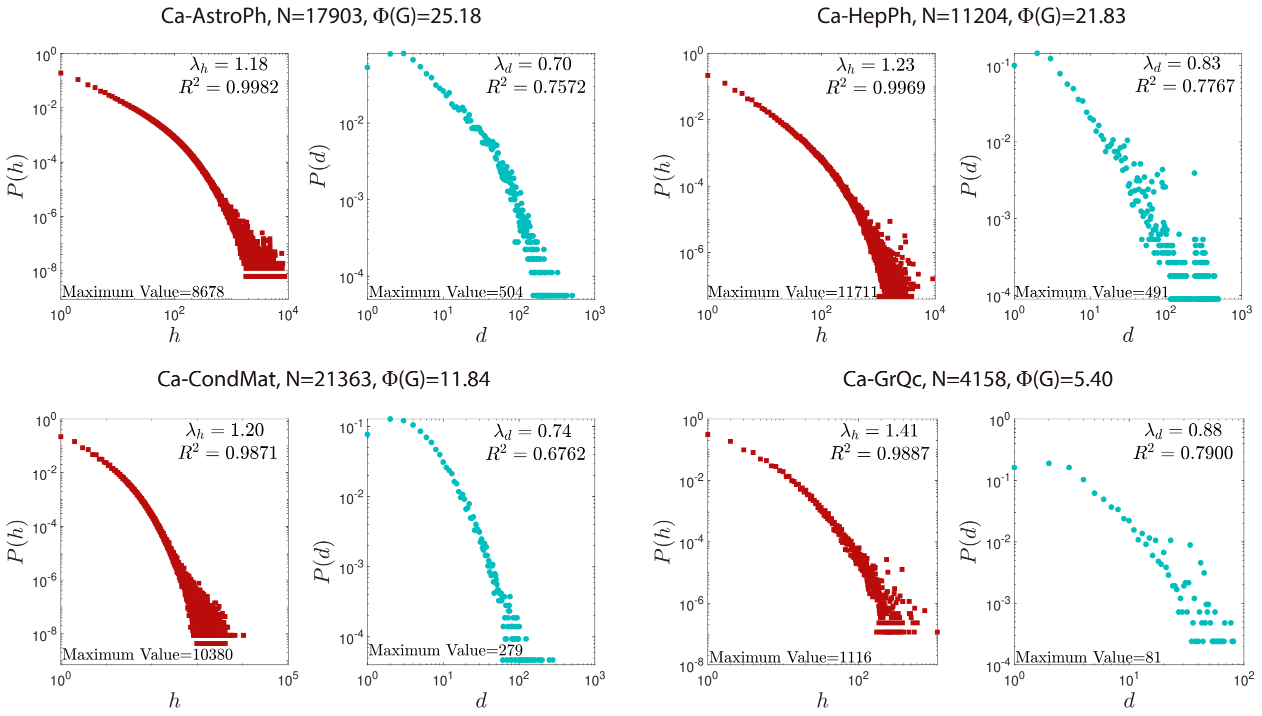

Fig. S7. PHA distributions $P(h)$ (red square) and degree distributions $P(d)$ (green circle) on log-log scales of collaboration networks. The goodness of fit R^2 and the maximum value of each distribution are also presented.

Web networks

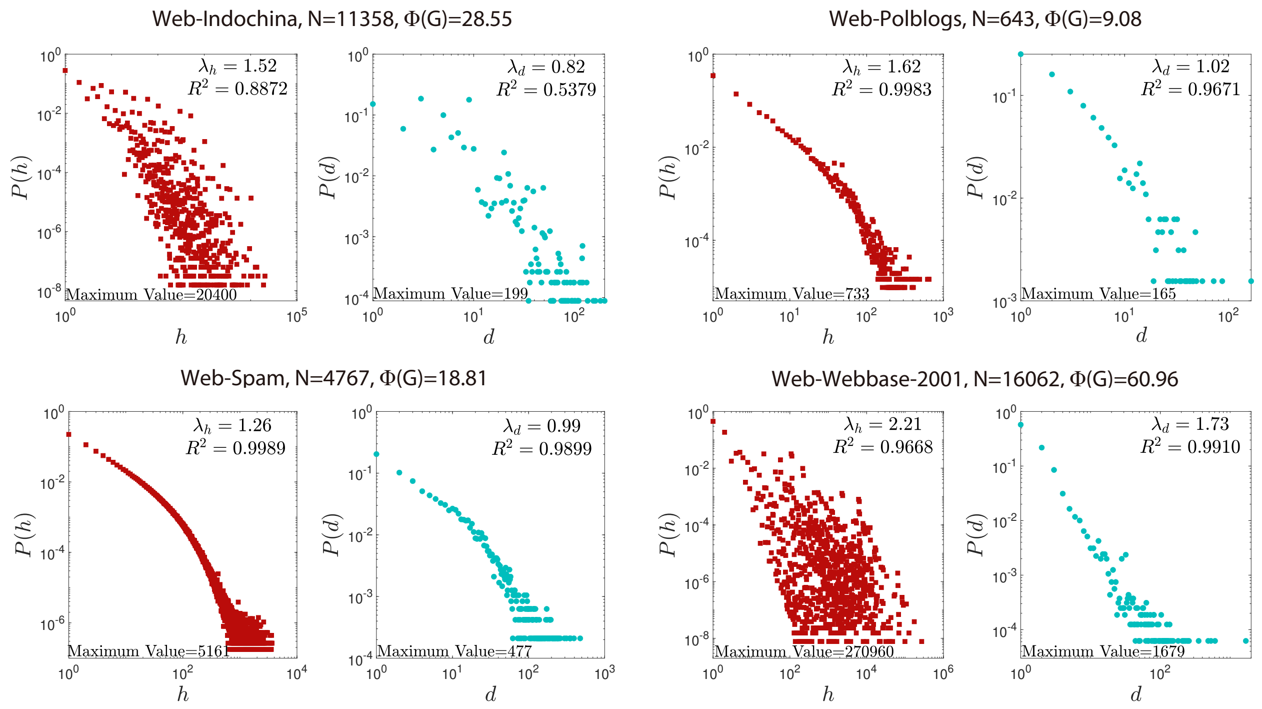

Fig. S8. PHA distributions $P(h)$ (red square) and degree distributions $P(d)$ (green circle) on log-log scales of web networks. The goodness of fit R^2 and the maximum value of each distribution are also presented.

No.	Name	Category	N	$< k >$	p_{edge}	L	C	r	$\Phi(G)$
1	bn-macaque-rhesus-brain-1	Brain network	242	25.2397	0.1047	2.2175	0.4501	-0.0548	11.0756
2	bn-fly-drosophila-medulla	Brain network	1770	10.1215	0.0057	2.9105	0.2645	-0.0943	4.1300
3	bn-mouse-retina-1	Brain network	1076	168.7937	0.1570	1.8613	0.5906	-0.1976	29.4874
4	bn-mouse-kasthuri-graph	Brain network	987	3.1125	0.0032	4.9136	0.0000	-0.2417	13.2047
5	power-bcspwr10	Power network	5300	3.1211	0.0006	20.8466	0.0880	-0.0527	6.5410
6	power-bcspwr09	Power network	1723	2.7789	0.0016	15.4879	0.0759	-0.1001	4.5706
7	US power	Power network	4941	2.6691	0.0005	18.9892	0.0801	0.0035	6.9937
8	power-685-bus	Power network	685	3.7431	0.0055	12.4221	0.1725	0.1812	7.2922
9	tech-WHOIS	Technological network	7476	15.2335	0.0020	3.5433	0.4889	-0.0425	17.3702
10	tech-as-caida2007	Technological network	26475	4.0326	0.0002	3.8756	0.2082	-0.1946	8.8364
11	tech-pgp	Technological network	10680	4.5536	0.0004	7.4855	0.2660	0.2382	11.6409
12	tech-routers-rf	Technological network	2113	6.2773	0.0030	4.6074	0.2464	0.0192	6.7771
13	econ-psmigr1	Economic network	3140	261.6439	0.0834	1.9241	0.4964	-0.1795	38.8944
14	econ-mahindas	Economic network	1258	11.9444	0.0095	3.5743	0.0613	-0.0622	30.4553
15	econ-orani678	Economic network	2529	68.6184	0.0271	2.4261	0.2741	-0.2797	71.3988
16	econ-poli-large	Economic network	15575	2.2431	0.0001	6.7123	0.0531	-0.1772	4.5668
17	bio-DR-CX	Biological network	3287	51.6518	0.0157	2.7809	0.1956	0.3436	27.7228
18	bio-SC-LC	Biological network	1999	20.4582	0.0102	3.2396	0.1665	0.1946	21.5007
19	bio-HS-LC	Biological network	4215	18.7317	0.0044	3.3721	0.1966	0.0629	32.9137
20	bio-grid-plant	Biological network	1272	4.2862	0.0034	8.2944	0.1676	0.0014	8.2150
21	fb-pages-tvshow	Facebook network	3892	8.8587	0.0023	6.2759	0.3737	0.5605	18.2799
22	fb-pages-food	Facebook network	620	6.7452	0.0109	5.0887	0.3309	-0.0322	9.0002
23	fb-pages-politician	Facebook network	5908	14.1185	0.0024	4.6641	0.3851	0.0182	18.9960
24	fb-pages-public-figure	Facebook network	11565	11.5933	0.0010	4.6230	0.1793	0.2020	25.4414
25	ca-AstroPh	Collaboration network	17903	22.0044	0.0012	4.1940	0.6328	0.2013	25.1806
26	ca-HepPh	Collaboration network	11204	20.9959	0.0019	4.6727	0.6216	0.6295	21.8267
27	ca-CondMat	Collaboration network	21363	8.5462	0.0004	5.3522	0.6417	0.1253	11.8434
28	ca-GrQc	Collaboration network	4158	6.4560	0.0016	6.0494	0.5569	0.6392	5.4007
29	web-indochina-2004	Web network	11358	8.3828	0.0007	6.4182	0.7099	0.1246	28.5535
30	web-polblogs	Web network	643	7.0918	0.0110	3.8281	0.2320	-0.2179	9.0880
31	web-spam	Web network	4767	15.6807	0.0033	3.7935	0.2860	0.0000	18.8184
32	web-webbase-2001	Web network	16062	3.1868	0.0002	10.2092	0.2243	-0.0985	60.9623

Table S1. Metadata of 32 real-world networks, where N is the number of nodes, $< k >$ is the average degree, p_{edge} is the edge density, L is the average shortest path length, C is the clustering coefficient, r is the assortativity coefficient and $\Phi(G)$ is the Path Hesitant Index.
